# Bilateral* Mycobacterium chelonae* Keratitis after Phacoemulsification Cataract Surgery

**DOI:** 10.1155/2017/6413160

**Published:** 2017-11-06

**Authors:** Jaime D. Martinez, Guillermo Amescua, Jesus Lozano-Cárdenas, Leejee H. Suh

**Affiliations:** ^1^Asociación Para Evitar la Ceguera, No. 46 Vicente García Torres, Delegación Coyoacán, 04030 México City, Mexico; ^2^Bascom Palmer Eye Institute, 900 N.W. 17th Street, Miami, FL 33136, USA; ^3^Harkness Eye Institute, Columbia University Medical Center, 635 West 165th Street, New York, NY 10032, USA

## Abstract

The purpose of this manuscript is to report the case of an 81-year-old patient who presented with bilateral keratitis after phacoemulsification surgery. Cultures came back positive for* Mycobacterium chelonae*. Despite aggressive topical and systemic antimicrobial treatment, the patient developed a corneal perforation in both eyes, treated with corneal glue in the right eye and corneoscleral patch in the left eye. After two years of follow-up, patient was free of infection in the right eye with visual acuity of 20/200 and the left eye progressed to phthisis bulbi. We present an unusual case of bilateral* Mycobacterium chelonae* keratitis associated with phacoemulsification cataract surgery. This case represents the importance of making clinicians aware of this devastating infection and highlights the need for better management to improve outcomes.

## 1. Introduction

The nontuberculous mycobacteria (NTM) species is composed of species other than the Mycobacterium tuberculosis complex, which consists of* Mycobacterium chelonae, Mycobacterium africanum*,* Mycobacterium bovis*,* and Mycobacterium leprae. Mycobacterium chelonae* belongs to* M. chelonae/abscessus *group, which are defined as those showing visible growth within 7 days on subculture on Lowenstein-Jensen medium, classified in the rapid growing nontuberculous mycobacteria (RGM) group. NTM are found in the environment in soil and drinking water. Animal reservoirs appear to be of lesser importance, although* M. chelonae* has been found in fish and frogs [1, 2].

Few cases of ocular infection after cataract surgery due to* M. chelonae *exist in the literature [3–11]. The reported cases of* M. chelonae* have been described after keratoplasty and following laser-assisted in situ keratomileusis (LASIK) surgery [2, 12, 13]. We present an unusual case of bilateral* Mycobacterium chelonae* keratitis associated with phacoemulsification cataract surgery to make clinicians aware of this devastating infection.

## 2. Case Report

An 81-year-old male was referred to the Bascom Palmer Eye Institute (BPEI) due to bilateral presumed infectious keratitis. The patient reported decreased vision and eye pain in both eyes one month after uncomplicated sequential phacoemulsification with posterior chamber intraocular lens implantation performed one week apart, in the right eye and then left eye, performed in South Florida. His medical history was only significant for chronic back pain with kyphosis. His past ocular history was only significant for nonneovascular age-related macular degeneration. There was no history of contact lens use. He was a retired driver and spent most of his time indoors.

**Figure 1:**
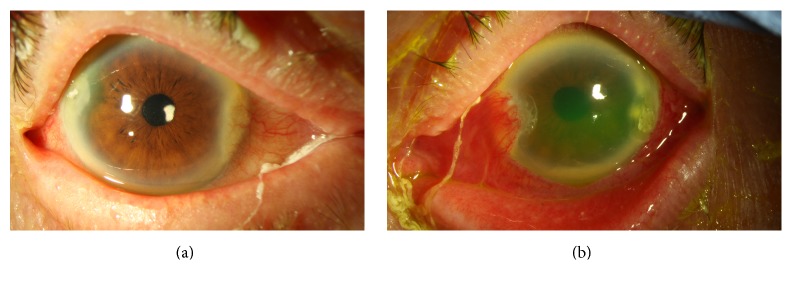
Slit-lamp photograph of the right eye (a) and left eye (b) demonstrates corneal stromal infiltrates localized at the temporal corneal lip and left eye with fluorescein staining of diffuse epithelia erosions and hypopyon.

On presentation, the patient was using a combination tobramycin/dexamethasone ointment 4 times a day and gatifloxacin 0.4% 6 times a day in both eyes. The best corrected-visual acuity (BCVA) on presentation was 20/80 in the right eye and 20/40 in the left eye. Intraocular pressure (IOP) was 11 mmHg in the right eye and 24 mmHg in the left eye. On slit-lamp examination, focal stromal infiltrates were noted near the temporal corneal incision in both eyes, associated with diffuse keratic precipitates (KP) with no epithelial defect. Anterior chamber examination revealed 3+ cells in the right eye and a 1 mm hypopyon in the left eye with an endothelial plaque underneath the temporal cataract incision. Both corneas wounds were Seidel negative (Figure 1). Posterior segment examination of the right eye showed no vitritis or posterior segment infection. Ultrasonography was performed but did not reveal signs of endophthalmitis. Corneal scrapings of both eyes were performed and sent for microscopic examination and cultures on chocolate agar, blood agar, Sabouraud agar, Thioglycollate, and blood culture bottles. Tobramycin and dexamethasone drops were discontinued and the patient was started on fortified vancomycin 50 mg/ml and gatifloxacin 0.4% every hour in both eyes and timolol 0.5% every 12 hours in the left eye. The next day, the acid-fast (AF) staining was reported as positive and vancomycin was replaced by compounded clarithromycin 1% drops along with gatifloxacin 0.4% every hour in both eyes.

**Figure 2:**
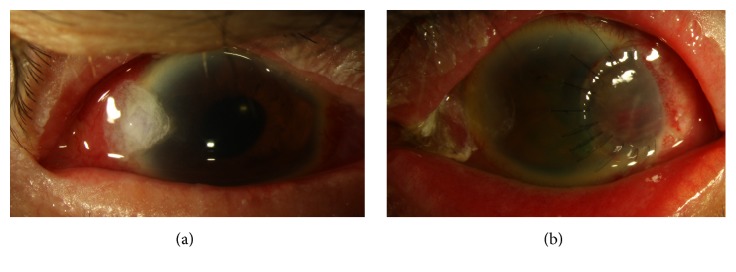
Slit-lamp photographs of the right eye (a) demonstrate a corneal glue and left eye (b) with corneal scleral patch graft.

A week later, the patient complained of increased pain and decreased vision in both eyes. Best corrected visual acuity was 20/400 in the right eye and counting fingers (CF) in the left eye. Intraocular pressure (IOP) was 6 mmHg in the right eye and 40 mmHg in the left eye. Slit-lamp examination of the right eye revealed Seidel positivity of the corneal incision and a flat anterior chamber. In the left eye, the stromal infiltrate was increased in size with corneal edema, and large KP; in the anterior chamber, a new hyphema and fibrinoid reaction were noted. The microbiology laboratory reported growth of* Mycobacterium chelonae*, with antimicrobial susceptibility to amikacin, moxifloxacin, and clarithromycin, but resistance to ciprofloxacin, doxycycline, and tobramycin. Cyanoacrylate glue was applied to the right eye and topical amikacin (8 mg/ml) was added to clarithromycin every hour in both eyes; dorzolamide and brimonidine were added to timolol in the left eye (Figure 2).

**Figure 3:**
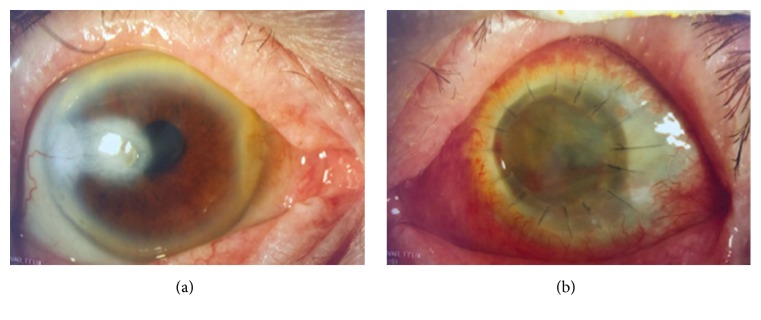
Slit-lamp photographs of the right eye (a) demonstrate paracentral corneal scar with no signs of infection and left eye (b) clear corneal graft with poor detail of anterior chamber.

Patient seemed to have poor compliance with therapy with poor family support. Despite the topical antimicrobial treatment, a month later the left cornea perforated and a corneal scleral patch graft was performed. Aqueous humor culture was negative (Figure 2). After surgery, cyclosporine 2% eye drops and azithromycin 500 mg PO twice a day were added to the patient's regimen. Four months from presentation, BCVA was 20/80 in the right eye and there was light perception (LP) in the left eye, with an IOP of 8 mmHg and 14 mmHg, respectively. Slit-lamp examination was significant for +1 bulbar injection in both eyes. There was temporal iridocorneal touch and mild stromal opacification around 11 o'clock in the right eye with a quiet anterior chamber. The left cornea had deep stromal neovascularization and an opaque corneal scleral patch (Figure 3). The patient was continued on clarithromycin and amikacin (8 mg/ml) drops four times a day in both eyes.

Two weeks later, the patient complained of worsening vision in the right eye with visual acuity of 20/200. He was noted to have a severe periocular contact dermatitis and was started on olopatadine 0.1% twice a day and the antibiotic regimen was changed to moxifloxacin every hour, dexamethasone/neomycin/polymyxin-b ointment 4 times a day, doxycycline 100 mg orally twice a day, and cyclosporine 2% 4 times a day. Corneal cultures at that time returned negative.

Nine months from presentation, BCVA was 20/400 in the right eye and LP in the left eye. Slit-lamp examination revealed a new epithelial defect in the right eye and corneal scraping returned positive for acid-fast bacteria. The patient was restarted on amikacin 8 mg/ml every hour while awake, olopatadine 0.1% twice a day, moxifloxacin four times a day, erythromycin four times a day, doxycycline 100 mg PO twice a day, and timolol eye drops twice a day.

Eleven months from presentation, ultrasound of the left eye showed mild to moderate vitreous opacities and an attached retina. A left penetrating keratoplasty (PK) was performed and a dense pupillary membrane was excised. The vitreous fluid was turbid and cultures returned positive for* Mycobacterium chelonae*. Intravitreal injections of amikacin and garamycin were therefore administered one time. Two months later, BCVA remained 20/400 in the right eye and there was no light perception (NLP) in the left eye. Slit-lamp examination of the right eye showed a paracentral scar with lipid keratopathy and new macular edema consistent with conversion to wet macular degeneration. The left eye demonstrated corneal edema and total retinal detachment. One dose of bevacizumab was given intravitreally in the right eye, which improved vision to 20/200. The patient has remained stable over the next two years with a visual acuity of 20/200 in right eye and NLP in left eye. Slit-lamp examination revealed a paracentral scar with no signs of inflammation and peripheral anterior synechia in the right eye; the left eye has become phthisical (Figure 3) (Table 1).

## 3. Discussion

We present an unusual case of bilateral* Mycobacterium chelonae* keratitis associated with phacoemulsification cataract surgery. Since 1989, there have been 10 patients reported in the literature who underwent cataract surgery and developed corneal wound infection by* M. Chelonae* [3–11]. Of the 10 cases, 3 of them were secondary to cataract surgery by phacoemulsification technique. Outcomes in all cases were infection-free at the last follow-up; 3 cases had penetrating keratoplasty (one case had 3 PKs). Visual outcomes of better than 20/200 were obtained in only two of the cases [3–11].

There have been reported cases with bilateral infectious keratitis after simultaneous bilateral laser in situ keratomileusis (LASIK) [14–17]. One case reported* Mycobacterium chelonae* infection with one eye resulting in a dense central corneal scar and the contralateral eye requiring PK.

Known risk factors to develop mycobacterial keratitis are trauma, ocular surgery, bad preocular tear film, inappropriate use of topical corticosteroids, systemic disease like diabetes mellitus, or cell mediated immunity disorders, epithelial defects, and contact lens use [10]. In our case it is difficult to know the exact risk factors that patient may be exposed to during or after cataract surgery. However, clinically the patient did not show any signs of persistent corneal epithelial defect or suture-related infections. M*ycobacterium* infection on self-sealing corneal incisions has been described in the literature [9, 18].

Nontuberculous mycobacterial (NTM) ocular infections are of special concern. Kheir et al. reported a systemic review of the literature with 174 case reports and case series with a total of 420 eyes infected with NTM and, by location, most cases reported infectious keratitis (290 eyes, 69%) followed by endophthalmitis (44 eyes, 10%), with most of them having a positive* M. chelonae* in 179 eyes (43%) and* M. fortuitum* in 62 (15%) eyes. In terms of antibiotic treatment in 192 eyes, amikacin alone (56 eyes, 29%) was used, followed by amikacin plus a macrolide antibiotic in 27 eyes (14%) [19]. Yamaguchi et al. reported a study of infectious keratitis outbreak after LASIK at a single laser center in Japan. A total of 39 eyes of 30 patients developed infectious keratitis, with the most common interval between LASIK and onset of infection being 2 weeks (36 eyes, 92%).* M. chelonae* was found to be responsible in 9 (23%) of the eyes, and topical amikacin, arbekacin, and erythromycin in addition to fourth generation fluoroquinolones were effective [20].

It is important to differentiate* Mycobacterium* specimens due to their differing susceptibility to different antibiotics. Commonly used agents against* M. chelonae* and* M. abscessus* include amikacin, imipenem and cefoxitin, ciprofloxacin, and ofloxacin (active against all groups except 75% of* M. chelonae*). Interestingly, doxycycline and minocycline were only active in 25% of* Mycobacterium chelonae* and resistant to* Mycobacterium abscessus* [21]. Our case cultures came back resistant to ciprofloxacin and doxycycline. In most cases reported in the literature, amikacin was the treatment of choice [3–9, 11]. However, there have been several reported cases of* M. chelonae* infection with resistance to amikacin, ciprofloxacin, and/or doxycycline antibiotics [5, 8, 10].

With the rise of use of topical fluoroquinolones for postoperative cataract surgery treatment, Hose et al. reported in an experimental cornea rabbit model with intrastromal inoculation of* M. chelonae* that therapy with gatifloxacin was more effective than ciprofloxacin alone and also demonstrated synergy in combination with fortified amikacin and clarithromycin [22]. There are successful clinical cases of patients with* M. chelonae* keratitis using fourth generation fluoroquinolones such as topical besifloxacin or gatifloxacin as an adjunct therapy [23, 24].

The use of clarithromycin has been recommended for cases that do not respond to amikacin. It is reported that clarithromycin is the oral drug of choice for treatment of cutaneous disease due to* Mycobacterium chelonae* [21]. This agent has a better penetration through intact epithelium [16]. Interestingly, despite using clarithromycin as an adjunct therapy, our patient's infection continued to progress. Mah-Sadorra et al. reported the use of oral clarithromycin in patient with* M. chelonae* keratitis after cataract surgery as a useful adjunct therapy [9, 10].

In general, patients with* M. chelonae* secondary to a cataract surgery had guarded outcome 7/10 (70%), with 2 of them having corneal graft and 6 cases with visual acuity being less than 20/800 [3–11]. Reviewing retrospectively our case and the literature, a take-home message is that we should be aggressive in dealing with this microorganism by starting treatment with multiple fortified antibiotics such as clarithromycin topical/oral, amikacin topical, and fluoroquinolones topical/oral. If patient has poor compliance, hospitalization until the infection is under control may be an option. Corneal collagen crosslinking (CXL) may be used as an adjunct therapy [25]. Photodynamic therapy with different light spectrum may be another option [26]. Early surgical intervention such as partial lamellar excisional keratectomy combined with focal cryotherapy may be a good option [27].

This case report provides to clinicians an overall outcome of patients diagnosed with* Mycobacterium chelonae* keratitis secondary to cataract surgery. Hopefully in the future new novel treatments will be developed to treat these complex cases. With such species, supervision with short time intervals, maintenance of adherence to therapy, and even hospitalization may be required to avoid catastrophic outcomes.

## Figures and Tables

**Table 1:** Summary of case report patient with *Mycobacterium chelonae* keratitis after cataract surgery.

Age (y)/sex	Procedure/eye	Culture results	Onset of signs and symptoms	Initial treatment	Culture yielded from and *in vitro* sensitivity	Postculture treatment	Steroid use	Surgical intervention/time from presentation	Final BCVA (Comment)
81 M	PHACO/PCIOL OU	*M. chelonae*	1 mo	Tobramycin dexamethasone and gatifloxacin 0.4% topical	Corneal scrape; sensitive: amikacin, moxifloxacin, clarithromycin resistance: ciprofloxacin, doxycycline, tobramycin	Clarithromycin 1% topical Amikacin (8 mg/ml) topical And gatifloxacin 0.4% topical	Yes Topical	(1) Corneal scleral Patch OS (1 mo) (2) PK OS (11 mo)	OD: 20/200 OS NLP; 2 y F/U OD infection free *OS failed corneal graft and TRD *

mo: months; y: years; M: male; PHACO: phacoemulsification; PCIOL: posterior chamber intraocular lens implantation; PK: penetrating keratoplasty; TRD: total retinal detachment; NLP: no light perception; bold letters: bad outcome.
